# The Impact of Framing on Acceptance of Cultured Meat

**DOI:** 10.3389/fnut.2019.00103

**Published:** 2019-07-03

**Authors:** Christopher Bryant, Courtney Dillard

**Affiliations:** ^1^Department of Psychology, University of Bath, Bath, United Kingdom; ^2^University Studies, Portland State University, Portland, OR, United States

**Keywords:** clean meat, cultured meat, cell-based meat, consumer psychology, framing

## Abstract

Cultured meat can be produced from growing animal cells *in-vitro* rather than as part of a living animal. This technology has the potential to address several of the major ethical, environmental, and public health concerns associated with conventional meat production. However, research has highlighted some consumer uncertainty regarding the concept. Although several studies have examined the media coverage of this new food technology, research linking different frames to differences in consumer attitudes is lacking. In an experimental study, we expose U.S. adults (*n* = 480) to one of three different frames on cultured meat: “societal benefits,” “high tech,” and “same meat.” We demonstrate that those who encounter cultured meat through the “high tech” frame have significantly more negative attitudes toward the concept, and are significantly less likely to consume it. Worryingly, this has been a very dominant frame in early media coverage of cultured meat. Whilst this is arguably inevitable, since its technologically advanced nature is what makes it newsworthy, we argue that this high tech framing may be causing consumers to develop more negative attitudes toward cultured meat than they otherwise might. Implications for producers and researchers are discussed.

## Introduction

### Framing

The ways in which humans strive to make sense of the world they inhabit has long been of interest to scholars in a variety of fields. Goffman (1) set the course for much of this research when he conceptualized framing as a “schemata of interpretation,” the manner by which humans organize information to make meaning both for themselves and others. Later research, especially in the fields of sociology and psychology, flushed out the way that frames work. Frames were seen as condensing reality, particularly in terms of fore-fronting certain aspects of reality, while back-dropping others (2–4). In the last four decades, an impressive body of literature on framing has developed in fields ranging from economics to cognitive linguistics (5–8).

Researchers in the various communication fields have focused their attention on the intentional use of frames, particularly in public life. Entman's well-known definition, “to frame is to select some aspects of a perceived reality and make them more salient in a communicating text, in such a way as to promote a particular problem definition, causal interpretation, moral evaluation, and/or treatment recommendation for the item described” [(9), p. 52] has undergirded and directed much of the research in this area. Frames have been investigated in terms of their role in media coverage, particularly news media (10, 11), political communication (12, 13) and advertising (14). One important distinction these scholars have sought to maintain is between the framing activities of those presenting information and those receiving it (15).

While interesting work has been done on the types of frames created by those presenting information (16–18), some of the most generative areas of research have been in terms of framing effects. This vein of research investigates how particular frames, often intentionally created, influence specific audiences (19, 20) and often seeks to establish frame effectiveness (21, 22).

Framing effects in terms of products and product features has more recently become a rich line of investigation. Work has been done on the type of frame employed and its effects in terms of willingness to pay, product preferences, and brand loyalty. For example, scholars have suggested that positive frames are generally more effective than negative ones, while allowing for the fact that there are occasions where a negative frame might be advantageous (23–25). Research has also focused on the effectiveness of marketing products in terms of social causes, particularly the environment. For example, Olsen et al. (26) found that while making green claims enhanced consumer favorability toward the brand, fewer claims rather than more were preferred. Cho (27) found that green frames worked best when they highlighted the consumer's own environmental impact. Ku et al. (28) noted that a consumer's motivations impacted how favorably they responded to green framing techniques.

Recent research in framing effectiveness has also demonstrated a growing curiosity around the role of images, whether stand alone or combined with text. Early theoretical research in this area (29, 30) made the case for the power of visuals, particularly in terms of emotional influence. Researchers have sought to examine this relationship in different contexts. For example, Iyer et al. (31) found that images of victims of the 2005 bombings in London elicited feelings of sympathy, while images of terrorists elicited feelings of fear and anger. Andrews et al. (32) found that cigarette packaging which included graphic images positively impacted young smokers determination to quit over an extended period of time.

Other scholars have taken an interest in the effects of multimodal frames, those which include a combination of texts and visuals. Geise and Baden (33) proposed a theoretical framework for understanding multimodal framing effects which draws attention to the amplifying effect of images. In terms of multimodal frames and products, recent work has suggested that textual framing might be more effective for some types of products, while visual framing or a combination of both works better for others (34, 35).

Of particular relevance here is the research on framing of genetically modified (GM) foods. Media coverage on GM foods has been shown to have a significant impact on public perceptions of, and behavior toward, the technology (36–39), and there is plenty of research on the nature of this coverage. Researchers have identified coverage on GM foods to be primarily driven by specific events such as food scares and environmental events (40, 41). Others have shown how mainstream media coverage diverges somewhat from scientific publications (42), and how stakeholders have been characterized to fit simple narratives (43). This demonstrates how media coverage is dependent on breaking stories, and how complexity is condensed for popular consumption.

Coverage has been different in different countries, however. Listerman (44) argued that, whilst US coverage of GM foods focused on the scientific-economic elements of the technology, German coverage was focused on the practical ethics and British coverage was focused on the public discourse. Coverage in the US was generally more positive than in the UK (41), and in China was universally positive or neutral (45). Whilst Botelho and Kurtz (40) argued that coverage within countries was fairly similar, Vicsek (46) noted that Hungarian coverage was particularly polarized. Interestingly, several researchers have commented on how genetic technology was generally framed much more negatively in relation to food than it was in relation to medicine within the same media outlets (38, 47, 48).

While there has been some important framing research concerning innovations in food products (49–51), there has been surprisingly little work on the intentional use of different frames to introduce audiences to new food products, particularly those closely connected to technological innovation. This article explores the effectiveness of different multimodal frames for a new food innovation, meat produced outside of an animal in a laboratory.

### Cultured Meat

In the near future, we will be able to produce meat directly from animal cells (52). Termed “cultured meat,” this technology will enable us to sustainably produce meat for a growing global population, whilst reducing animal suffering on an enormous scale (53, 54). However, research into public perceptions of cultured meat has indicated that some consumers may have reservations around the concept (55).

Although many consumers recognize the potential ethical and environmental benefits of cultured meat, some have concerns about its alleged unnaturalness, which can lead to concerns about food safety (56–58). Recent studies have demonstrated how these perceptions can be invoked or avoided by different framings.

The Good Food Institute (59, 60) has given substantial attention to the question of what cultured meat should be called, demonstrating that consumers are significantly more likely to find “clean meat” appealing than other names including “cultured meat” and “cell-based meat.” This finding has been replicated by Bryant and Barnett (61). Siegrist et al. (57), meanwhile, have demonstrated that less technical descriptions of cultured meat lead to higher consumer acceptance compared to more technical descriptions.

These findings are relevant for the interpretation of much of the existing research on cultured meat. For instance, Verbeke et al. (58) noted many consumers in their focus groups reacted with disgust to the concept and perceiving few personal benefits—yet, these responses were undoubtedly influenced by the video participants were shown, which describes “synthetic meat” being grown in labs. Likewise, Laestadius and Caldwell (62) conducted an analysis of online comments on news stories about cultured meat, but note “…the framing of the issue in each individual article may have influenced perceptions of [cultured meat]” (p. 2466).

Therefore, the framing of cultured meat is likely to have a substantial impact on consumer perceptions, though this has yet to be studied empirically (55). Whilst Goodwin and Shoulders (63) reported that European and American media coverage of cultured meat commonly discusses its benefits, production process, timescale, history, and skeptics, Dilworth and McGregor (64) identified naturalness as a key focus in Australian print media. Indeed, stories about cultured meat frequently feature “science themed” photos such as meat in a petri dish in a lab [e.g., (65, 66)]. Meanwhile, Hopkins (67) has commented that coverage in western media has focused disproportionately on the reactions of vegetarians.

While a variety of frames pertaining to cultured meat are available, little is known about how they may affect consumer attitudes. A wealth of existing research indicates that frames have an impact on public attitudes, but this has not yet been formally studied in the context of cultured meat. The present study seeks to understand how different frames affect consumer attitudes, beliefs, and behavioral intentions toward cultured meat.

## Methods

We used an experimental survey to test the effect of different framings of cultured meat on consumer attitudes, beliefs, and behavioral intentions. This study received ethical approval from the Portland State University Institutional Review Board.

### Participants

Participants were U.S. adults recruited through Amazon MTurk, a microtasking platform frequently used in social research. MTurk enables researchers to get high quality affordable data from a sample which is more representative than college samples which have commonly been used in the past (68). However, we did find evidence of some illegitimate or duplicate responses. After removing these responses, the sample size dropped from 527 to 480. Participants were each paid $0.50 for their time.

The demographic breakdown of participants is shown in Table 1:

**Table 1:** Demographic breakdown of participants.

		**Number**	**Percentage**
Gender	Male	276	57.5
	Female	202	42.1
	Other	2	0.4
Age	18–25	92	19.2
	26–35	229	47.7
	36–45	84	17.5
	46–55	38	7.9
	Over 55	37	7.7
Region	Northeast	109	22.7
	South	185	38.5
	Midwest	81	16.9
	West	105	21.9
Diet	Omnivore	422	87.9
	Pescatarian	35	7.3
	Vegetarian	14	2.9
	Vegan	9	1.9

As shown here, the sample is slightly skewed toward younger age groups (in particular 26–35) and toward males. The south of the country is also slightly over-represented, though overall the sample is reasonably representative.

### Procedure

First, participants read some information about the study and gave their consent to take part. They were then asked for demographic information, including gender, age group, region, and which foods they eat. These foods were later used to determine diet.

Next, participants indicated whether they had heard of cultured meat before. They then read the following description of cultured meat:

“Clean meat (also called cultured meat or *in-vitro* meat) is real meat which is grown from animal cells without the need to raise animals. It should not be confused with meat substitutes such as soy, since it is real animal meat it has the same taste, texture, and the same or better nutritional content as conventionally-produced meat.”

Next, participants gave one word that they first thought of when they thought about cultured meat. This was an open question, and was later used to identify illegitimate responses. Participants also indicated how familiar they were with cultured meat on a 5-point Likert scale (1 = Not at all familiar, 5 = very familiar).

Participants were then allocated to one of three experimental conditions. These conditions (see Table 2) contained an image and a short piece of text. They corresponded to three different framings that cultured meat could be presented in.

**Table 2:** Text and images presented to participants in each condition.

**Societal benefits**	**High-tech**	**Same meat**
Clean meat has many benefits for society like reducing harm to the environment and helping animals. 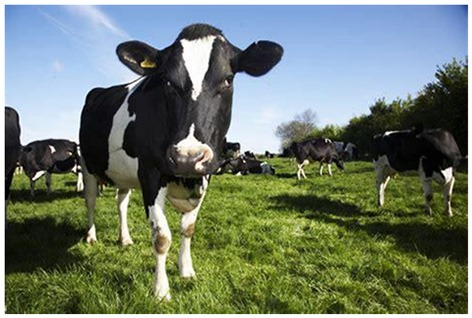	Clean meat is made using highly advanced technology in a state of the art laboratory. 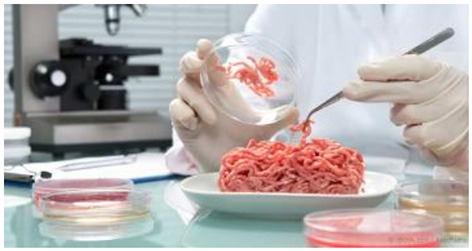	Clean meat tastes like conventional meat, is increasingly affordable and can be healthier to eat. 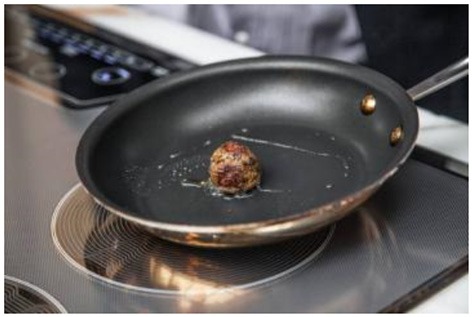

Next, participants were asked to rate their attitude toward cultured meat on a 5-point Likert scale (1 = Very favorable, 5 = Very unfavorable).

Participants were then asked to rate their agreement with five statements about cultured meat on 5-point Likert scales (1 = Strongly disagree, 5 = Strongly agree). The statements were about cultured meat's healthiness, safety, environmental friendliness, sensory quality, and benefits for society. Next, participants rated four concerns about cultured meat using 5-point Likert scales (1 = Not at all concerned, 5 = Extremely concerned). The concerns were about cost, taste, naturalness, and safety. These are common concerns and benefits identified by Bryant and Barnett (55).

Finally, participants rated their willingness to eat cultured meat using 5-point Likert scales (1 = Definitely yes, 5 = Definitely No). Participants were asked about their willingness to try cultured meat, willingness to buy cultured meat regularly, willingness to eat cultured meat as a replacement for conventionally produced meat, and willingness to eat cultured meat compared to plant-based meat substitutes. These measures were adapted from Wilks and Phillips (69).

During analysis, we removed 47 illegitimate or duplicate responses. We also computed diet based on foods which participants said they ate. Finally, we recalibrated all Likert scales such that higher numbers represented more positive opinions of cultured meat. This involved reverse coding the attitude rating, concern ratings, and behavioral intentions ratings.

### Experimental Design

We opted for an experimental design whereby participants would see one of three framings before answering questions about cultured meat. This approach is fairly common in similar research (57, 70) as it allows for direct comparison between groups who have seen different information. While some authors (71) have used repeated measures designs (before/after information), we decided to avoid this approach since participants might be anchored to responses they give before reading additional information. Indeed, Bekker et al. (71) implemented a Solomon four-group design to rule out such effects.

These three framings were chosen because they represent common discourses on cultured meat. Potential societal benefits, the technical scientific nature of the product, and the sensory similarity to conventional meat are all themes which occur in media coverage of the topic (62). Furthermore, they are well-defined and distinct from one another in that they foreground a different aspect of the technology, and could therefore be expected to produce different perceptions to some extent.

It is worth noting that we did not include a control group as such. We could have asked a control group about their perceptions of cultured meat after reading basic facts about the product with no framing. However, such a presentation of information is unlikely to occur in the media. Moreover, one could argue that there is no such thing as “no framing” in this context—any information we could give about cultured meat would, by definition, focus on some aspects more than others, and therefore would frame the product in some way. Therefore, we decided not to include a control group in the conventional sense.

It is also worth noting that some measures (e.g., about taste, healthiness, and benefits to society) asked about things which were explicitly mentioned in some of the experimental manipulations. For example, the “same meat” framing mentions that “Clean meat tastes like conventional meat,” and we might therefore expect responses to reflect this. We should bear in mind the content of the messages when interpreting the results; higher agreement with statements about aspects of the technology mentioned in the descriptions is to be expected, and can be taken as confirmation that participants have engaged with and believed the material. Of course, this may not be the case, and beliefs about specific aspects of the technology may not be sensitive to such information if it is not deemed credible.

## Results

### Overall Findings

Before examining differences between experimental groups, we looked at the findings across all experimental conditions. Our findings are comparable to those observed in previous U.S. studies: we found that 64.6% of participants were probably or definitely willing to try cultured meat, which is very similar to the rates observed in previous research (69, 70). Only 18.4% were probably or definitely not willing to try cultured meat, whilst 16.9% were unsure.

Similarly optimistic rates were found with regards to participants' willingness to buy cultured meat regularly (49.1% were probably or definitely willing to do this; 24.5% were probably or definitely not willing to; 26.4% were undecided) and willingness to eat cultured meat as a replacement for conventional meat (48.5% were probably or definitely willing to do this; 26.6% were probably or definitely not willing to; 24.9% were undecided). Of the 243 participants who currently ate plant-based meat substitutes, 49.8% were somewhat or much more likely to eat cultured meat; 25.5% were somewhat or much less likely, and 24.7% were undecided.

Overall, this indicates a fairly high willingness to eat cultured meat regardless of framing, with almost two thirds of participants being willing to try it, and almost half willing to buy it regularly and eat it instead of conventional meat. This indicates a substantial potential market for cultured meat, and provides evidence that cultured meat could displace a considerable amount of demand for conventional meat.

#### Demographic Variations in Acceptance

Previous research has discussed demographic variations in acceptance of clean meat, and some studies have found higher acceptance amongst men, younger people, and omnivores [see (55)]. To test for significant differences in acceptance between demographic groups, we conducted a series of three one-way between-group ANOVAs with gender, age, region, and diet as independent variables, and the range of acceptance measures as dependent variables. No significant differences were found between respondents from different regions.

In terms of gender, we detected several significant differences between men and women. In line with previous research, men had more positive views of cultured meat than women, on average. These differences were significant with respect to attitude, perceived safety, perceived taste, perceived benefits for society, willingness to try, willingness to buy regularly, willingness to replace conventional meat, and willingness to eat over plant-based alternatives (*p* < 0.05). However, men were more concerned about the cost compared to women (*p* = 0.01).

Age was also a factor which affected views on cultured meat. Younger people generally had more positive views than older people, with a steady decline in attitudes in older age groups. Curiously, the 56+ age group was an exception here—people in this group tended to have more positive views than those in the 36–45 and 46–55 age groups. Significant differences were found in the different age groups' attitudes, perceived taste, perceived benefits for society, willingness to try, willingness to buy regularly, willingness to replace conventional meat, and willingness to eat compared to plant-based alternatives (*p* < 0.05).

Participants with different diets also had differing views on cultured meat. We observed interesting differences between vegetarians/vegans and those who eat meat/fish. Vegetarians/vegans were significantly less willing to try cultured meat than meat/fish-eaters (*p* = 0.014) and significantly less willing to eat cultured meat compared to plant-based alternatives (*p* = 0.01), but meat/fish-eaters had significantly higher concerns about the taste, naturalness, and safety of the product (*p* < 0.05). This probably reflects a relative lack concern on the part of vegetarians/vegans, who were not intending to eat the product anyway. This partly reflects the findings of Wilks and Phillips (69), who similarly found vegetarians/vegans to be more positive about some aspects of cultured meat, but relatively unwilling to eat it themselves.

#### Word Associations

Participants gave word associations immediately after learning about cultured meat. Word associations is a technique which has been used in previous research to explore consumer perceptions of novel products (61, 72). A codebook was developed based on common categories which the word associations fit into. Each word was then categorized independently by both researchers. We agreed on the categories of 83.5% of the words; the remaining words were categorized after consultation between the researchers. The categories of words given by consumers are shown in Table 3.

**Table 3:** Word associations given by participants after learning about cultured meat.

**Category**	**No. of words**	**Percentage**	**Example words**
Artificial	73	15.2	Fake, unnatural, artificial
Science	54	11.3	Scientific, laboratory, chemicals
Positive	50	10.4	Good, awesome, super
Natural	40	8.3	Natural, no hormones, unprocessed
Unusual	35	7.3	Weird, strange, different
Food	27	5.6	Beef, calories, steak
Healthy	26	5.4	Fat-free, healthy, good for health
Clean	25	5.2	Sterilized, washed, soap
Disgust	24	5.0	Disgusting, yuck, gross
Other	18	3.8	Options, jars, grown
Taste	16	3.3	Tasty, bland, delicious
Food technology	14	2.9	GMOs, cultured meat, laboratory meat
Interesting	12	2.5	Interesting, intriguing
Animals	10	2.1	Chicken, fish, pig
Ethical	10	2.1	Ethical, cruelty-free, humane
Fear	10	2.1	Unsafe, danger, creepy
Negative	9	1.9	Abomination, dystopia, never
Safety	7	1.5	Safe, safety, passes regulation
Uncertainty	7	1.5	Confusing, why, unobtainable
Environment	5	1.0	Sustainable, biofriendly, green
Special diet	5	1.0	Vegetarian, Halal, Kosher
Cost	3	0.6	Expensive, pricey, cost
Total	480	100	

### Experimental Findings

Before proceeding with analysis, we wanted to verify that key demographic and familiarity variables associated with cultured meat acceptance had been evenly distributed across experimental conditions. To this end, we tested for significant differences between experimental groups using Chi square and ANOVA tests as appropriate.

Chi square tests reveal that there are no significant differences between conditions in the proportions of participants in each gender (χ^2^ = 4.009, *p* = 0.405), age group (χ^2^ = 8.762, *p* = 0.363), region (χ^2^ = 6.726, *p* = 0.347), or diet (χ^2^ = 10.463, *p* = 0.106). ANOVA tests reveal no significant differences between conditions in the proportion of participants who had heard of cultured meat [*F*_(2, 477)_ = 1.530, *p* = 0.218] or the familiarity with cultured meat [*F*_(2, 477)_ = 0.895, *p* = 0.409]. Given no significant differences between experimental conditions with respect to these variables, we can rule this out as a source of bias.

#### Attitudes and Beliefs

We tested for significant differences in attitudes and beliefs between experimental conditions using one-way ANOVA analyses. The results (shown in Table 4) indicate several significant differences (*p* < 0.05) between experimental conditions, indicating that the framing had a statistically significant effect on key attitudes and beliefs about cultured meat.

**Table 4:** ANOVAs showing differences between experimental conditions in attitudes and beliefs.

**Variable**	**ANOVA (2, 477)**	**Societal benefits M (σ)**	**High tech M (σ)**	**Same meat M (σ)**
Attitude	*F* = 5.711, *p* = 0.004	3.45_a_ (1.13)	3.11_b_ (1.32)	3.55_a_ (1.20)
Belief that cultured meat is healthy	*F* = 5.093, *p* = 0.007	3.43_ab_ (0.98)	3.23_b_ (1.12)	3.60_a_ (1.00)
Belief that cultured meat is safe	*F* = 3.247, *p* = 0.040	3.56_ab_ (1.08)	3.40_b_ (1.12)	3.71_a_ (1.01)
Belief that cultured meat is good for the environment	*F* = 3.336, *p* = 0.036	3.98_a_ (0.99)	3.40_b_ (1.08)	3.97_a_ (0.94)
Belief that cultured meat tastes the same as conventional meat	*F* = 3.003, *p* = 0.051	3.27_a_ (1.07)	3.40_ab_ (1.08)	3.56_b_ (1.06)
Belief that cultured meat has benefits for society	*F* = 0.760, *p* = 0.468	3.70_a_ (1.02)	3.63_a_ (1.08)	3.78_a_ (1.02)
Concern about cost	*F* = 0.935, *p* = 0.393	2.70_a_ (1.19)	2.53_a_ (1.09)	2.57_a_ (1.19)
Concern about taste	*F* = 0.534, *p* = 0.587	2.38_a_ (1.05)	2.26_a_ (1.06)	2.36_a_ (1.22)
Concern about naturalness	*F* = 2.055, *p* = 0.129	2.40_a_ (1.19)	2.14_a_ (1.18)	2.36_a_ (1.24)
Concern about safety	*F* = 1.064, *p* = 0.346	2.15_a_ (1.15)	1.99_a_ (1.15)	2.16_a_ (1.16)

Within rows, mean values which are significantly different using Tukey's HSD (*p* < 0.05) are denoted using different subscript letters. Values which share a subscript letter are not significantly different.

As shown here, the experimentally manipulated framing had a statistically significant effect on attitude, belief that cultured meat is healthy, belief that cultured meat is safe, and belief that cultured meat is good for the environment (although no pairwise comparisons were significantly different for the latter variable). Conversely, although the omnibus ANOVA showed no significant effect on the belief that cultured meat tastes the same as conventional meat, *post-hoc* tests did show a significant pairwise difference. No significant differences were found on the belief that cultured meat has benefits for society, or on any measures of concern about cost, taste, naturalness, or safety.

In each case, the “same meat” framing was shown to be conducive to the most positive attitudes, whereas the “high tech” framing was shown to be conducive to the least positive attitudes.

#### Behavioral Intentions

Next, we tested for significant differences between framings in behavioral intentions using a one-way ANOVA. A similar pattern of results emerges with respect to behavioral intentions, as shown in Table 5.

**Table 5:** ANOVAs showing differences between experimental conditions in behavioral intentions.

**Variable**	**ANOVA (2, 477)**	**Societal benefits M (σ)**	**High tech M (σ)**	**Same meat M (σ)**
Willingness to try cultured meat	*F* = 9.808, *p* < 0.001	3.79_a_ (1.10)	3.30_b_ (1.55)	3.85_a_ (1.62)
Willingness to eat cultured meat regularly	*F* = 7.313, *p* = 0.001	3.50_a_ (1.10)	3.03_b_ (1.33)	3.48_a_ (1.21)
Willingness to replace conventional meat	*F* = 5.488, *p* = 0.004	3.37_a_ (1.16)	3.03_b_ (1.36)	3.49_a_ (1.24)
Willingness to eat compared to plant-based meat substitutes	*F* = 4.834, *p* = 0.008	3.42_ab_ (1.20)	3.10_b_ (1.27)	3.51_a_ (1.23)

Again, participants who saw the “high tech” framing were significantly less willing to try cultured meat, buy cultured meat regularly, eat cultured meat as a replacement for conventional meat, and eat cultured meat compared to plant-based meat substitutes compared to those who saw other framings.

Although these differences were significant, the effect sizes were relatively small. It should be noted that perceptions of cultured meat are likely to be changed by further information, and may not be stable over time.

## Discussion and Conclusion

In this study, we demonstrated that the framing of cultured meat has a significant effect on many attitudes and beliefs about the product, as well as behavioral intentions toward it. Our results somewhat mirror the findings of Siegrist et al. (57), who found that more technical descriptions of cultured meat lead to lower acceptance compared to less technical descriptions. This is probably because the information in the “high tech” condition (particularly the image) were evocative of an image of science and unnaturalness. Siegrist and Sütterlin (73) demonstrated that perceived naturalness of cultured meat mediated the acceptability of risk.

### Implications

These findings offer important insight for those publicizing and promoting cultured meat. While more research is clearly needed in terms of the frames currently used both by companies in the industry and the media, existing work suggests that the most common frame used thus far may be the least effective in garnering consumer acceptance. As noted previously, many of the media reports have featured images like the petri dish and used terminology like “test tube meat” to introduce this concept and the products associated with it to the public. While fledgling ventures might welcome media interest and the benefits associated with earned media, these findings suggest that the frames favored by the media might do more harm than good. At the same time, this must be weighed against the benefits of increased consumer familiarity (55). Since more familiar consumers are more likely to say they would eat cultured meat, it may be the case that any coverage is better than no coverage, regardless of framing.

The findings may also inform future decisions for the messaging of this product, once the products are close to launching and dedicated advertising and marketing campaigns are underway. A quick perusal of comments by company executives, venture capitalists and supporting institutions in this area suggest a laudable commitment to transparency in terms of the production process. The outcomes of the research here argue for a high level of intentionality in how the process is shared with the public. Perhaps the most effective approach would be to have that information readily available for consumers who seek it, but not to have the high tech process as the dominant frame in promotional materials. Instead, producers should consider shifting their frame from discussing the production process to discussing product features and societal benefits. This should be done both in terms of paid and earned media activities.

Whilst producers and traditional media outlets have a certain degree of control over what framings are employed in discussions of cultured meat, social media represents a domain in which such control is substantially limited. Fellenor et al. (74) have demonstrated how social media, compared to traditional media, can lead to substantially different framings, with certain groups selecting and emphasizing different “frame fragments” (p. 1174) as they share information. As the authors comment, the curated nature of social media news feeds can lead to individuals having different aspects of a concept highlighted or backdropped. In this context, this may lead to a variety of personalized frames. Notably, such frames are likely outside the control of cultured meat producers and traditional media sources. The same is true of those developed through other unconventional media such as blogs.

### Contributions to the Field

This article contributes to the field in several important ways. First, it advances the conversation on multimodal frames through its consideration of responses to image and text combinations. As these combinations reflect the type of messaging that most consumers are exposed to in contemporary marketing and promotional efforts, it deepens understanding of consumer reactions in contexts with a variety of messaging modes. Second, this article contributes to the growing field of research on very new products (VNP) and specifically the marketing of products associated with advanced technological processes. As more and more of these types of products are introduced into the marketplace, it is important for the field to further develop a focus on consumer responses to them Finally, and perhaps most importantly, this research offers a noteworthy addition to a fledgling but growing area of interest in a wide host of issues surrounding the food technology of cultured meat. It complements work done by Goodwin and Shoulders (63) and Dilworth and McGregor (64) who identified varied media frames of cultured meat in different countries and offers an invitation for additional research in this area. Indeed, stories about cultured meat frequently feature “science themed” photos similar to the one used in the process framing condition here [e.g., (65, 66)]. As this product moves through the concept phase to the production process and finally to market, researchers in a wide host of disciplines can make significant contributions not only to their fields of study, but also to society as they explore this transformative technology.

### Limitations

There are several limitations to acknowledge here. Firstly, the data is subject to well-known concerns about the quality of self-reported data. Data which is self-reported rather than observed is likely to be biased in some predictable ways; participants may report their past behaviors inaccurately due to poor memory, or their intended behaviors may not represent what they actually do due to poor forecasting. Moreover, some participants may give socially desirable answers, particularly when the subject is moralized, potentially leading them to over-report their intention to eat cultured meat in this case.

Secondly, we have some concerns about the data quality. Data was collected from Amazon MTurk, which has recently been subject to concerns about bots answering surveys (75). Indeed, we identified 47 responses which seemed not to be genuine (most had given nonsensical answers to text input questions) but it is difficult to know whether more went unnoticed. This is likely to be a problem for any researchers using online survey response platforms, and such problems have recently been well documented with MTurk.

Finally, the external validity of an online study which asks participants about a future product is inevitably limited. Whilst we gave all participants information about cultured meat, it is possible that this information would be interpreted differently in the context of taking an online survey compared to making actual purchase decisions in a restaurant or store. Indeed, seeing just an image and a strapline may be a contrived way to consume information, although arguably this could be similar to a headline and image in media.

Overall, there are some concerns about data quality and the external validity of the survey, however these are minor concerns and we have taken steps to mitigate these where possible.

### Future Research

Future research on the topic of framing new technologies could explore how producers attempt to influence media frames, how successful they are in promoting their preferred frames, and the downstream effect on consumer attitudes. Systematically comparing the frames used by producers with those present in media reports using content analysis could highlight which aspects of reality each choose to foreground. This will be particularly relevant to other consumer technologies which may become available imminently, and which can be readily interpreted in different ways, for example self-driving vehicles.

In terms of consumer research in relation to cultured meat specifically, the field would benefit from rigorous content analyses of frames used by both producers and the media over the last 5–7 years. What are the dominant frames presented to consumers both by producers through their own promotional materials like YouTube videos and by journalists in their stories? Have these frames changed over time? Do these frames differ from those which occur on social media? And finally, how are consumer perceptions and intentions influenced by the frames they encounter and have these changed over time?

Future research on cultured meat acceptance, meanwhile, could attempt to track consumer attitudes over time. Such a longitudinal design could allow researchers to attempt to observe the real effect of relevant news on consumer attitudes. Observing shifts in specific beliefs and attitudes could provide a way to observe the changes that take place when consumer attitudes shift over time, and could provide a method for measuring the master frame through which consumers interpret cultured meat. Moreover, it would be able to test the idea that acceptance will increase over time as people become more familiar with the product and products become commercially available.

Finally, further exploration of public opinions of cultured meat on social media and blogs may be warranted. As we have discussed, social media may lead to a variety of personalized frames which are outside the control of producers and traditional media outlets. Such an environment could lead to further insights about important narratives about cultured meat as they develop.

## Data Availability

The participants in this study were not asked for permission to share the data publicly. Therefore, the dataset for this study is not available.

## Ethics Statement

The study received ethical approval from the Portland State University Institutional Review Board. Participants indicated their consent to take part as part of the online survey process. The study was of a general population, no vulnerable participants were specifically recruited.

## Author Contributions

CD and CB: research design, survey instrument, writing manuscript, and editing manuscript. CD: ethics application and data collection. CB: data analysis.

### Conflict of Interest Statement

CB is the Director of Social Science at the Cellular Agriculture Society, which aims to promote cellular agriculture. The remaining author declares that the research was conducted in the absence of any commercial or financial relationships that could be construed as a potential conflict of interest.
